# Anatomical basis of erector spinae plane block: a dissection and histotopographic pilot study

**DOI:** 10.1007/s00540-020-02881-w

**Published:** 2020-12-19

**Authors:** Daniele Bonvicini, Rafael Boscolo-Berto, Alessandro De Cassai, Michele Negrello, Veronica Macchi, Ivo Tiberio, Annalisa Boscolo, Raffaele De Caro, Andrea Porzionato

**Affiliations:** 1 Department of Urgency and Emergency, Anesthesiology and Intensive Care Unit, University-Hospital of Padova, Padua, Italy; 2 Department of Neurosciences, Institute of Human Anatomy, University of Padova, Via A. Gabelli 65, 35127 Padua, Italy; 3 Veneto Region Reference Center for the Preservation and Use of Gifted Bodies, Veneto Region, Padua, Italy; 4 Anesthesia and Intensive Care Unit, University-Hospital of Padova, Padua, Italy

**Keywords:** Erector spinae plane block, ESP, Histotopography, Cadaveric study

## Abstract

**Purpose:**

Erector spinae plane (ESP) block is an interfascial blockade used in different clinical scenarios. This study investigated the ventral extent of dye diffusion in ESP block.

**Methods:**

The ultrasound-guided ESP block was bilaterally performed with an injection at the T5 vertebral level (21-Gauge, 50 mm needle), using diluted black tissue marking dye (20 mL; 1:4 ratio with standard saline solution) instead of local anesthetic on two fresh-frozen corpses within the body donation program of the University of Padova. Subsequently, the gross anatomical dissection was performed by a combined posterior plus anterior approach, and the histotopographic examination completed.

**Results:**

Macroscopically by gross anatomical dissection, the dye spreading ranged on the dorsal side of the chest from T2/3 to T10/11 with an extension up to 10 cm laterally, and on the ventral side of the chest from T2/3–T9/10. Microscopically by histotopographic examination, the dye diffused ventrally to the intercostal spaces (2–3 and 5–6 spaces on the right and left, respectively) by following the blood vessels coupled to the dorsal nerve passing through the costotransverse foramen.

**Conclusions:**

The anterior pathway of dye diffusion from the site of injection within the erector spinae muscle group during an ESP block seems to follow the blood vessels and dorsal rami of spinal nerves, suggesting the passing through the costotransverse foramen to reach the anterior paravertebral space and the intercostal nerves. These findings display an anterior histotopographic diffusion of dye resembling a paravertebral block.

**Supplementary Information:**

The online version contains supplementary material available at 10.1007/s00540-020-02881-w.

## Introduction

Erector spinae plane (ESP) block is an interfascial blockade, first described by Forero in 2016 [1]. It consists of an injection of the local anesthetic in a fascial plane placed between erector spinae muscles and the tip of the transverse vertebral process [1]. The anesthetic spreads over the fascial plane both in the cranial and caudal direction, also diffusing anteriorly and laterally at several levels by one dermatome per 3.4 mL of injected liquid [2]. It provides analgesia in a wide range of different clinical scenarios [3, 4].

However, the effectiveness of the ESP block is not entirely reliable and reproducible [3], and some questions still remain unaddressed. The ongoing debate is mainly focused on the real spread of anesthetics, as by anatomical dissections, radiological imaging, and paravertebral endoscopy, some authors described conflicting evidence on the issue. Sometimes, no spreading to the anterior paravertebral space was reported at all both in humans and pigs [5–8], or on the contrary, the spread to the neural foramina [1, 9, 10], to the anterior paravertebral space and intercostal nerves [1, 9–13], or the epidural space was mentioned [9, 13]. All the anatomical studies reported a wide dye spread on the dorsal rami [1, 5–7, 9–14].

On these bases, the present study was aimed to investigate the spread of the dye to the anterior paravertebral space and its pathway of diffusion, by both an anatomical morphometric and histologic study on corpses.

## Materials and methods

Two fresh-frozen corpses within the body donation program “Donation to Science” of Padua University [15] were used for the present study. All the procedures performed in this study involved human bodies from the Veneto Region Reference Center for the preservation and use of gifted bodies (Deliberation of the Regional Council of the Veneto Region No. 245, Mar 8th, 2019; No. 389897), in accordance with the national laws, and the ethical standards of the regional/national research committees, as well as with the 1964 Helsinki declaration and its later amendments or comparable ethical standards. Written informed consent was provided to join the body donation program.

The donors were a 78 year old male affected by atrial fibrillation (Corpse A), and a 93 year old female affected by mild hypertension (Corpse B), both died of heart disease. The sorted donors had no significant past clinical condition affecting the present study. In particular, they were not long-term bedridden, which would have potentially led to changes in the trophism and integrity of the posterior muscle planes due to the prolonged supine position.

The experimental procedure and dissection were compliant with a dedicated internal protocol implemented to standardize the operative steps.

### The erector spinae plane block

The ESP block was performed coherently to daily clinical practice [1, 4] by an anesthesiologist expert in performing regional anesthesia (DB). The procedure was performed on both sides of the chest.

Each corpse was placed in a prone position and a preliminarily local ultrasound examination excluded any deviation of chest structures from the expected normal human anatomy, at the same time confirming that the relevant ultrasound anatomy was consistent with what is observed clinically, in the livings.

The spinous processes were palpated and marked directly on the skin by a dermographic pencil, and the correctness of the final marking was confirmed by sonographic inspection. A 21-gauge, 50 mm needle (Pajunk Sonoplex, PAJUNK Medizintechnologie GmbH, Geisingen, Germany) was inserted with a cephalad-to-caudal direction into the posterior thoracic wall at the T5 level, to reach the respective transverse process.

The proper needle tip positioning was checked by ultrasound guidance with a 12.5 MHz linear probe (SonoSite M-Turbo, FUJIFILM Sonosite, Inc., Bothell, WA, USA): the visualization of a linear fluid spread that distended the fascial plane between the erector spinae muscles group and the transverse process while injecting 2 mL of normal saline solution was considered confirmatory.

**Fig. 1:**
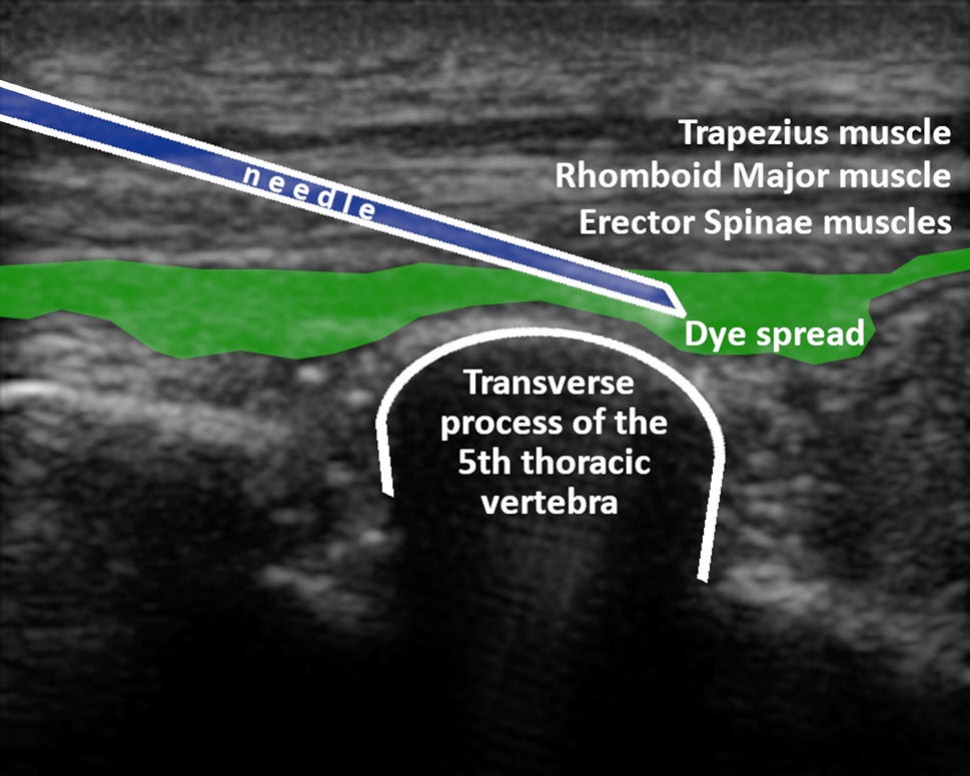
Ultrasound image related to the erector spinae plane block procedure. The visualization of a linear fluid spread that distended the fascial plane between the erector spinae muscles group and the transverse process while injecting 20 mL of diluted Black tissue marking dye confirmed the proper needle tip positioning.

Subsequently, 20 mL of black tissue marking dye (01DIA250, Diapath S.p.A., Martinengo-Bergamo, Italy) diluted by normal saline solution with a 1:4 ratio were injected on each side, while monitoring by ultrasound for its spread along the anatomical plane between the erector spinae and the transverse processes [1] (Fig. 1; Online Resource 1). This black tissue marking dye was chosen, because it endures the Hematoxylin-Eosin staining.

### Gross anatomical dissection

The gross anatomical dissection was performed 20 min after the ESP block, using a posterior midline incision corresponding to the spinous processes from above C7 to the lower lumbar vertebrae (RBB, AP). The skin was overturned laterally to expose the posterior thoracic wall appropriately. The superficial muscles (i.e., trapezius, latissimus dorsi, and rhomboids), the underlying erector spinae muscles (i.e., iliocostalis, longissimus, and spinalis) enveloped by the thoracolumbar fascia, and the deepest muscles of the posterior wall (i.e., multifidus thoracis, rotatores thoracis breves and longi, levatores costarum breves and longi, and thoracic intertrasversarii) were in turn identified and removed by detaching. At this stage, the extent of dye spread was evaluated both in craniocaudal and in the mediolateral direction. The craniocaudal spreading of dye was described concerning the vertebral levels and their correspondent ribs, and this was explored from first to twelfth thoracic vertebrae. At each vertebral level, the sidelong diffusion was measured from the apex of the corresponding transverse vertebral process to the point of maximum lateral extension.

**Fig. 2:**
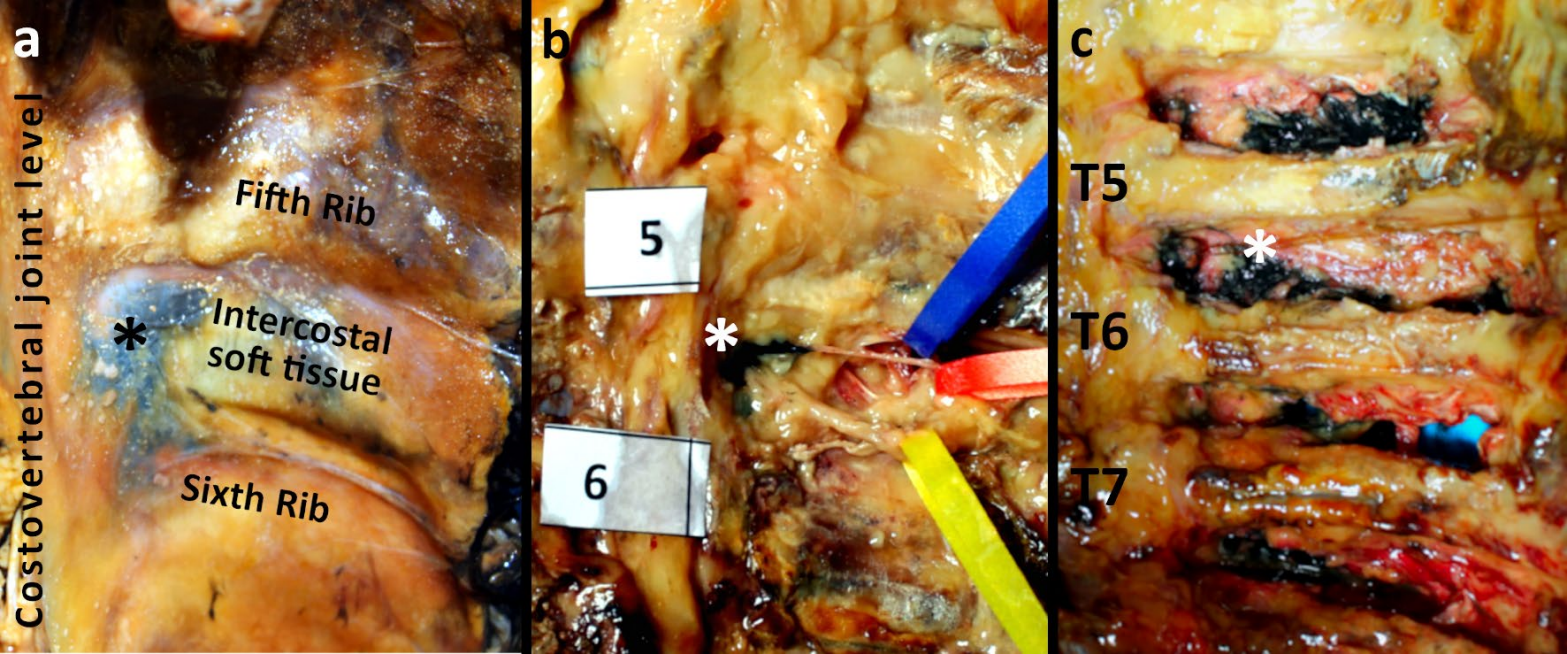
Ventral macroscopic dye spread after erector spinae plane block. **a** Corpse B—ventral view. Parietal pleura in place. The dye (asterisk) involved the intercostal soft tissue at the T5/6 level of the anterior paravertebral space, on the left side. **b** Corpse B—ventral view. The dye (asterisk) involved the intercostal soft tissue at the intervertebral foramen of the T5/6 level on the left side. The blue stripe holds the intercostal vein; the red stripe holds the intercostal artery; the yellow stripe holds the fifth intercostal nerve. **c** Corpse B—ventral view. The dye involved the intercostal soft tissue at the intervertebral foramen from T4/5 to the T7/8 level on the left side. The fifth intercostal nerve (asterisk) was dissected and suspended with a suture thread for demonstration purposes. Note that while in frame “a” the ink appeared superficially only at level T5/6, in frame “c” it was detected from level T4/5–T7/8, due to an in-depth dissection of the intercostal soft tissues up to the intervertebral foramina.

**Fig. 3:**
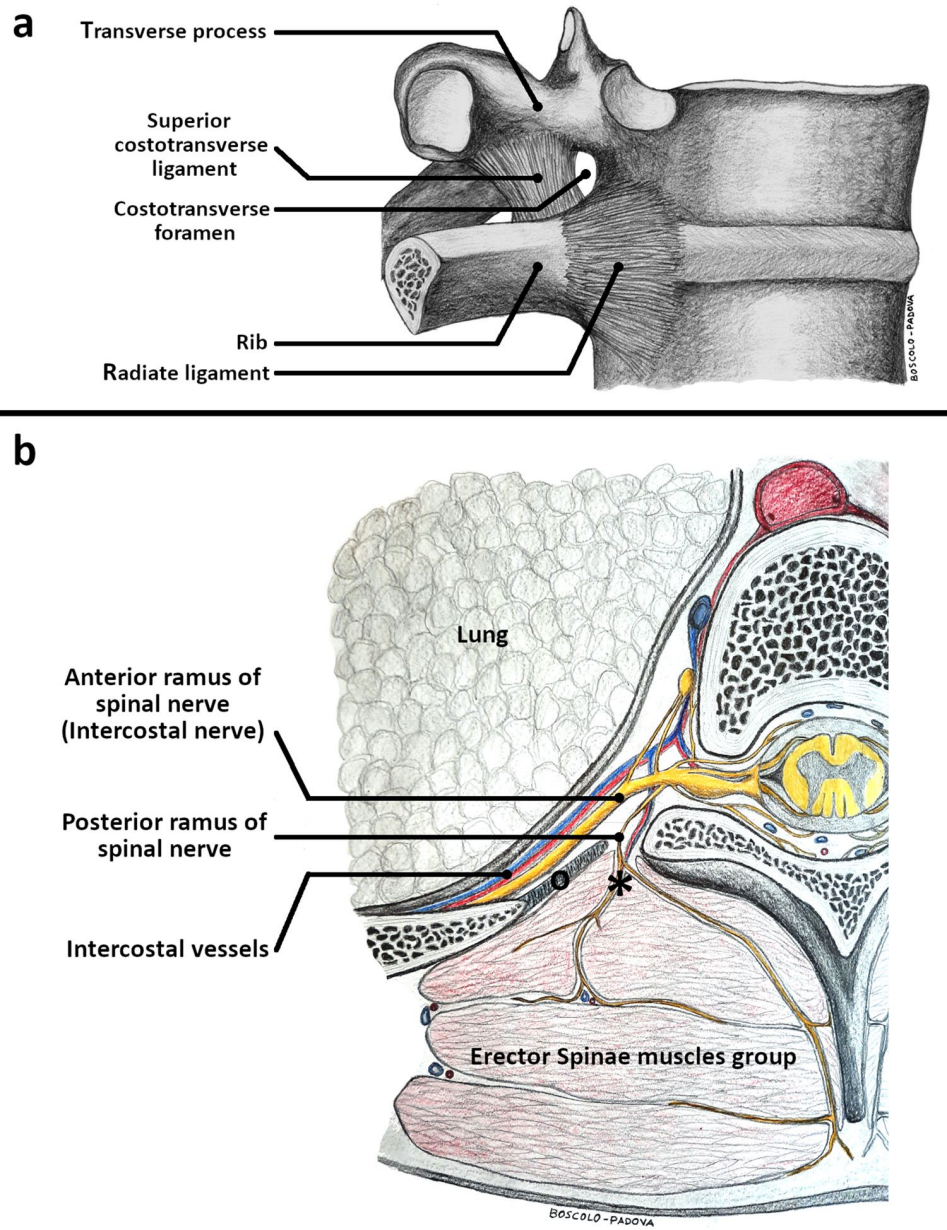
The costotransverse foramen and its content. **a** The intercostal tissue between two adjacent ribs includes the internal intercostal membrane deep to the external intercostal muscles, the external intercostal muscles, the internal intercostal muscles, and the innermost intercostal muscles, variably allocated as a function of the sagittal plane explored. Its medial portion is placed anteriorly to the costotransverse foramen, and is made up of loose connective tissue surrounding the intercostal vessels and nerves. The costotransverse foramen is an anatomical space bordered cranially by the transverse process, caudally by the underlying rib, laterally by the medial free edge of the superior costotransverse ligament, and medially by the lamina and facet joints. **b** Cross-section of the chest at the T5/6 level. The intercostal soft tissue encompasses the intercostal nerve and intercostal vessels. The dorsal ramus of the spinal nerve and its accompanying blood vessels pass through the costotransverse foramen (asterisk), medially to the superior costotransverse ligament (o), to reach the erector spinae muscles group.

Subsequently, the spine was disjointed at the level of C5–C6 and L1–L2, the sternoclavicular joint was disconnected, and the ribs from the first to the twelfth were dissected at the posterior axillary line using a costotome. The costovertebral whole was preserved. Ventrally, after removal of the parietal pleura, each intercostal space was taken jointly with the anterior paravertebral soft tissue as a whole from the costal angle to the intervertebral foramen. The harvested intercostal soft tissue corresponds macroscopically to what is shown in (Fig. 2). It is placed between two adjacent ribs, in front of the transverse process and the costotransverse ligaments (Fig. 3). As a consequence, its medial part is placed anteriorly to the costotransverse foramen. The intercostal nerve and intercostal vessels are included in the intercostal soft tissue (Fig. 3). Macroscopic inspection of the intervertebral foramen was afterwards performed to detect the presence of the black tissue marking dye.

### Histotopographic examination

**Fig. 4:**
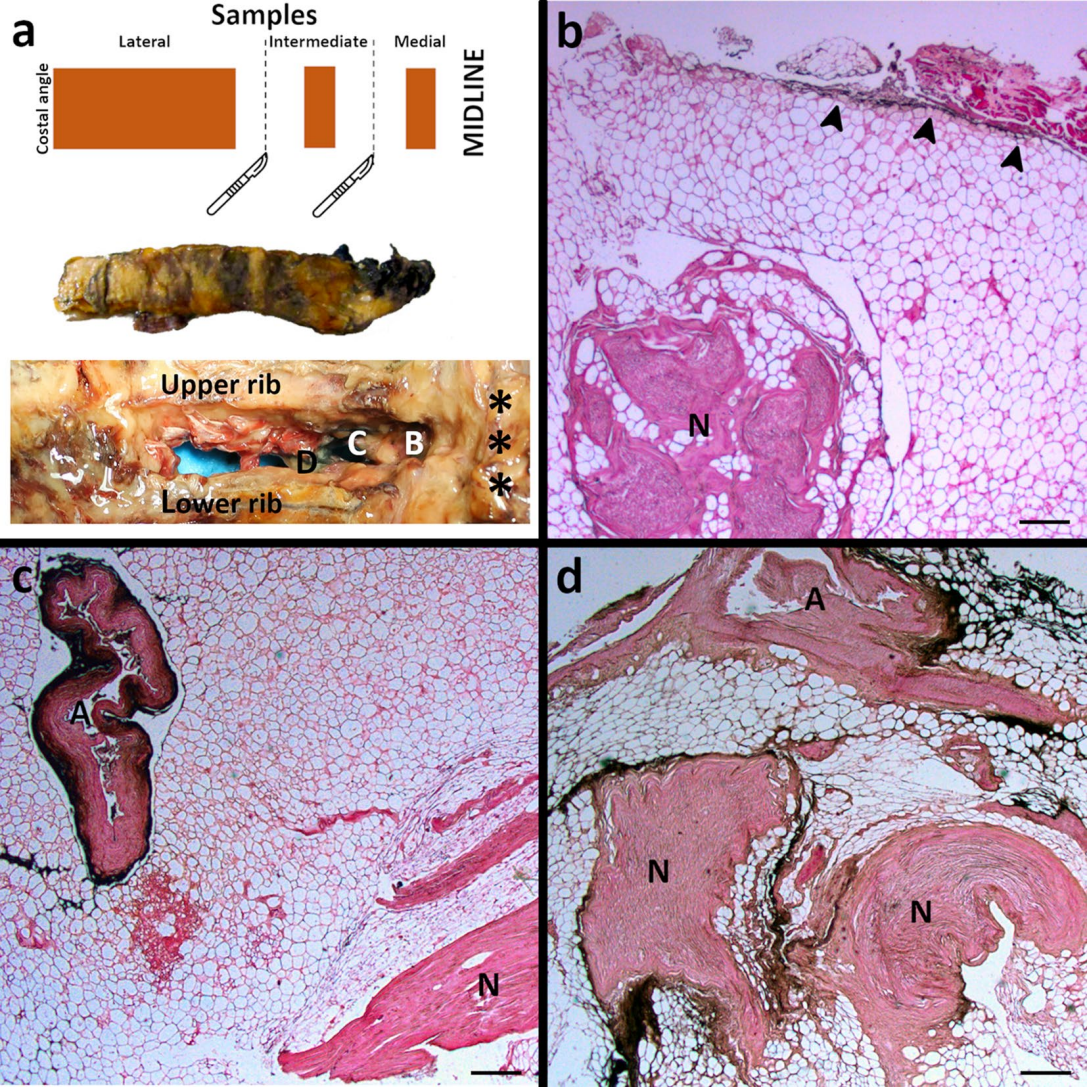
Cutting layout and histotopographic examination of intercostal soft tissue. **a** The upper part shows the cutting scheme to obtain the medial, intermediate and lateral samples from each harvested intercostal soft tissue. The middle part shows the harvested intercostal soft tissue fixed in a 10% formalin solution for 30 days. The medial portion is the closest to the costovertebral joint level. The intermediate and lateral portions are placed more laterally, towards the costal angle. The lower part shows the picking point of the harvested intercostal soft tissue, between the upper and lower rib of a chosen level. Asterisks identify the costovertebral joint level. The intervertebral foramen (B), the costotransverse foramen (C), and the superior costotransverse ligament (D) are shown. The cutting scheme, the harvested intercostal soft tissue, and its picking point are corresponding, from top to bottom. **b** Weakly positive (+), with presence of black tissue marking dye in the adipose tissue (arrows), but at a distance from the vascular and nervous structures (N). Hematoxylin-Eosin (H and E) stain, scale bar is equal to 300 µm. **c** Moderately positive (++), with presence of black tissue marking dye in the adventitia of an artery (A) but not near the nerve (N). Hematoxylin-Eosin (H and E) stain, scale bar is equal to 300 µm. **d** Strongly positive (+++), with presence of black tissue marking dye on the nerve surface (N). An artery with colored adventitia is also shown (A). Hematoxylin-Eosin (H and E) stain, scale bar is equal to 300 µm.

Histological examination was aimed at analyzing the presence and pattern of diffusion of the black tissue marking dye into the retrieved specimens, with particular reference to the nerve, vein, and artery. All the specimens were mounted on plastic cases to avoid deformation artefacts and fixed in a 10% formalin solution for 30 days. For technical convenience, three samples (medial, intermediate, and lateral) were obtained from each specimen through sequential cuts orthogonal to the major axis, as shown in Fig. 4. The medial portion was the closest to the costovertebral joint level. The intermediate and lateral portions were placed more laterally.

**Fig. 5:**
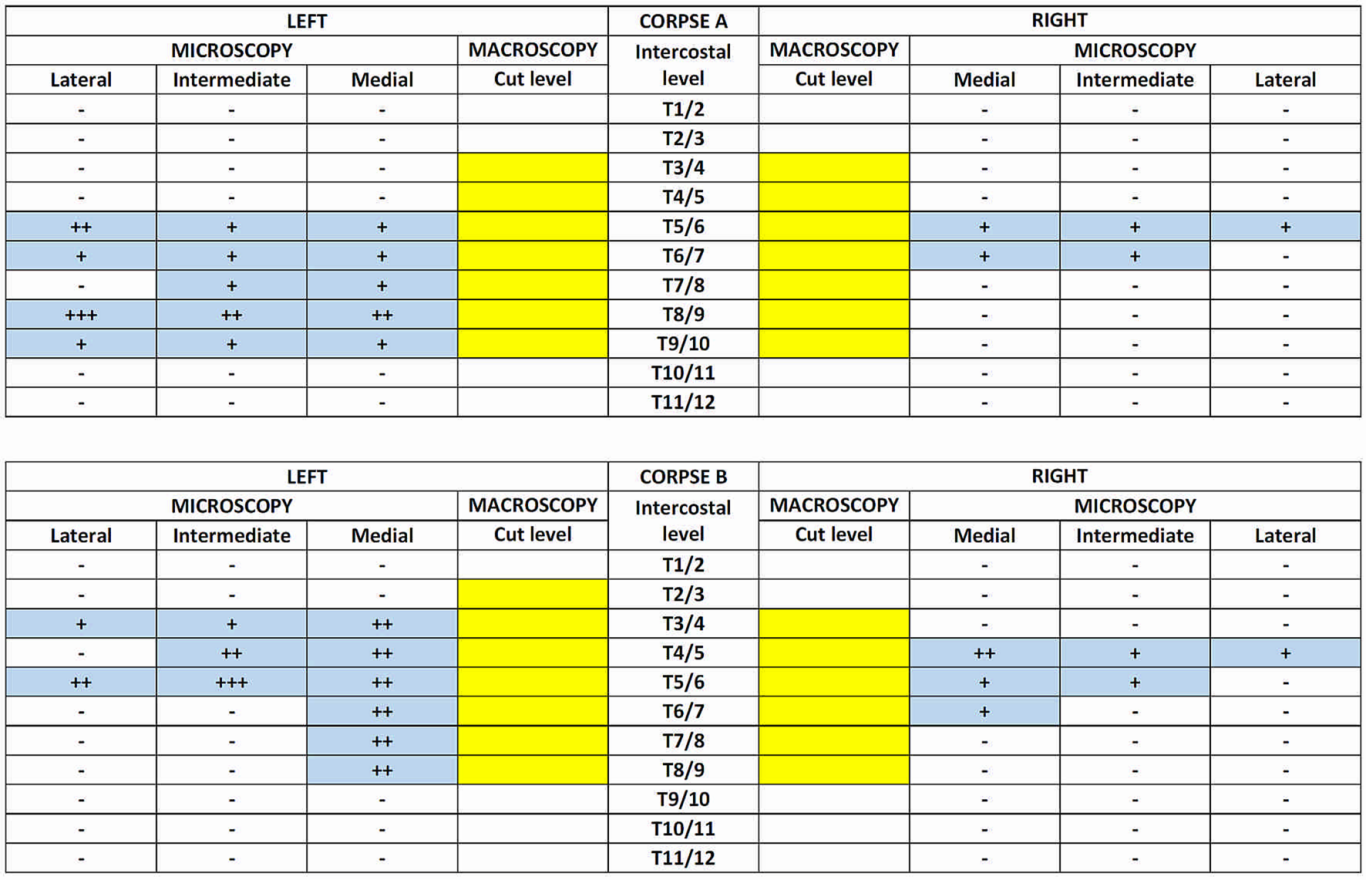
Synoptic table of macroscopic and microscopic dye distribution on dissected chests. Macroscopic synopsis. Craniocaudal dye spreading is expressed as the involvement (yellow colored box) or not (blank box) of a given intercostal thoracic level by macroscopic evaluation of the staining involving the most medial portion of sampled intercostal soft tissues, or filling the intervertebral foramen. Microscopic synopsis. Craniocaudal dye spreading is expressed as the involvement (blue colored box) or not (blank box) of a given intercostal thoracic level (TX/Y) by histotopographic examination. The involvement of an intercostal thoracic level was assessed with reference to the intercostal space between two following thoracic vertebrae, namely TX and TY. The coloring was graded as follows. (−) Negative: absence of black tissue marking dye on the microscopic anatomical preparation; (+) Weakly positive: presence of black tissue marking dye in the adipose tissue, but at a distance from the vascular and nervous structures; (++) Moderately positive: presence of black tissue marking dye nearby the adventitia of the vessels but not near the nerve; (+++) Strongly positive: Presence of black tissue marking dye next to the nerve.

The samples were then paraffin-embedded and serially sectioned (5 μm thickness) from one side to the other. One every ten sections was stained with Hematoxylin–Eosin and independently analyzed by two authors (AP and RBB) with particular reference to the presence/diffusion of the black tissue marking dye into the sample, with or without the involvement of the intercostal nerve, vein and artery (Fig. 4). The samples only showed a progressive alpha-numerical code applied by the technical staff who performed the staining. No element directly traceable to the origin of the sample was recognizable during the histotopographic evaluation. Therefore, the blind evaluation was carried out according to the following graduation of the possible positivity found on the microscopic anatomical preparation (Figs. 5, 4):

(−) Negative: Absence of black tissue marking dye on the microscopic anatomical preparation.

(+) Weakly positive: Presence of black tissue marking dye in the adipose tissue, but at a distance from the vascular and nervous structures.

(+ +) Moderately positive: Presence of black tissue marking dye near the adventitia of the vessels but not nearby the nerve.

(+ + +) Strongly positive: Presence of black tissue marking dye next to the nerve.

Disagreements of the classification were settled by discussion, basing the consensus on the simultaneous re-evaluation of the microscopic preparation, whereas persisting discrepancies were solved by evaluating additional sections next to the debated one.

## Results

**Fig. 6:**
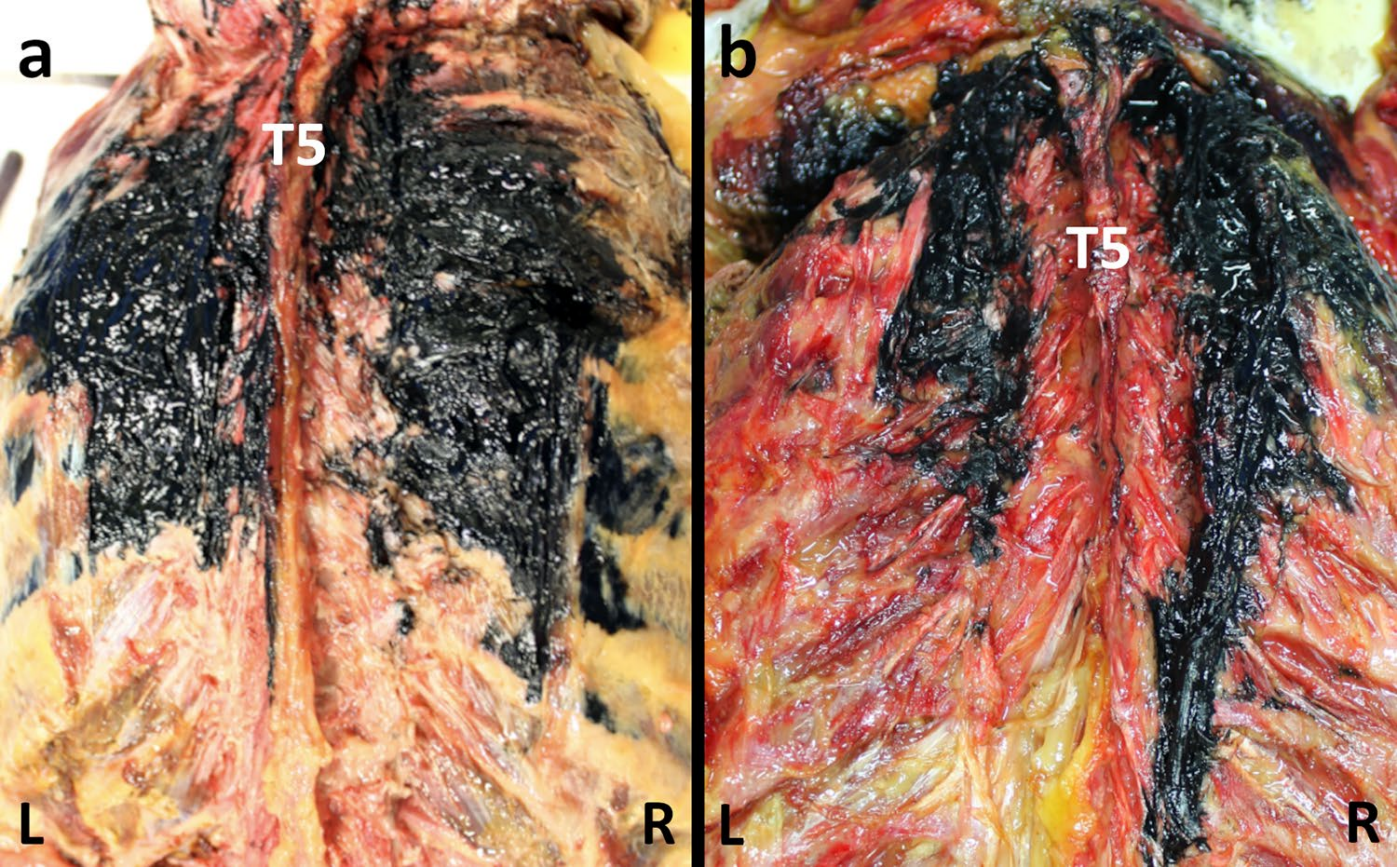
Dorsal macroscopic dye spread after erector spinae plane block. **a** Corpse A—dorsal view. The dye spread ranged from T2/3–T9/10 both on the right and left side, with a lateral spread reaching the maximal extension (9 cm) at the costal angle from T5/6–T7/8 level to progressively narrow at the contiguous levels. *T5* fifth thoracic level, *R* right side, *L* left side. **b** Corpse B—dorsal view. The dye spread ranged on the left side from T2/3–T8/9, and the right side from T2/3–T10/11, with a lateral spread reaching the maximal extension (10 cm) at the costal angle of T7/8 level to progressively narrow at the contiguous levels. *T5* fifth thoracic level, *R* right side, *L* left side.

**Fig. 7:**
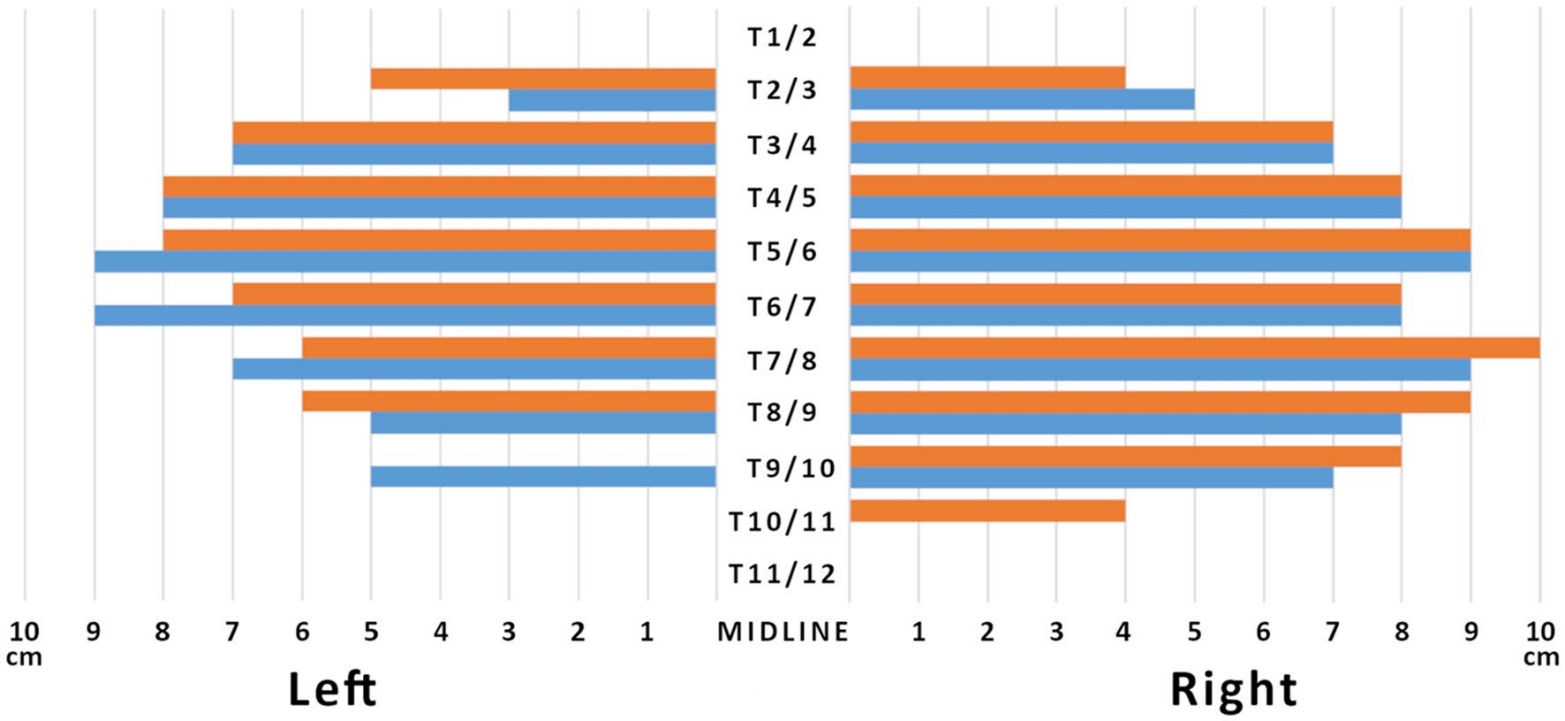
Synoptic table of macroscopic dye distribution on dissected chests. Dorsal view. Craniocaudal dye spreading is expressed as the involvement (colored) or not (blank) of a given thoracic level. Blue bars are referred to corpse A, Orange bars are referred to corpse B. The involvement of a given thoracic level was assessed with reference to the corresponding vertebral body. The sidelong diffusion was measured from the apex of the corresponding transverse vertebral process to the point of maximum lateral extension.

Four sides from two corpses were injected according to an ESP block and subsequently dissected. The cadaveric section did not reveal the significant presence of the dye in the subcutaneous tissue, except for minor leakage along the injection pathway. Posteriorly, there was extensive dye spread observed in planes both superficial and deep to the erector spinae muscles group and the deepest muscles of the posterior wall (multifidus thoracis, rotatores thoracis breves and longi, levatores costarum breves and longi, and thoracic intertrasversarii). The craniocaudal spread of the dye was over many segments and differed across the muscular planes, as much as for left or right sides. Macroscopically, once removed all the dorsal muscles as mentioned above, the dye spread ranged on the left side from T2/3–T9/10 and T2/3–T8/9, on the right side from T2/3–T9/10 and T2/3–T10/11 for corpse A and B, respectively (Figs. 6, 7). Overall, a significant lateral spread was documented as reaching the maximal extension (9–10 cm) at the costal angle of T5/6–T8/9 level to progressively narrow at the contiguous levels  (Figs. 6, 7).

On the ventral surface of the chest, the dye was detected at the intervertebral foramen on the left side from the level T3/4–T9/10 and T2/3–T8/9 for corpse A and B, respectively, showing an extension comparable as for the dorsal surface  (Figs. 5, 7). On the right side, the dye spreading was narrower than on the dorsal surface, ranging from the level T3/4–T9/10 and T3/4–T8/9 for corpse A and B, respectively (Fig. 5).

Microscopically, the presence of dye on the left side levels resembled its macroscopic distribution for both corpses. However, while for the corpse A, the staining generally involved only the adipose tissue at a distance from the vascular and nervous structures, being noted near the adventitia of the intercostal vessels or nerve only at the levels T5/6 (lateral sample) and T8/9 (whole specimen); for corpse B, the dye near the adventitia of the vessels was almost ubiquitous for the positive samples, with evidence for staining next to the intercostal nerve at T5/6 (intermediate sample) level, and the only adipose tissue involvement for the intermediate and lateral samples of T3/4 level (Figs. 4, 5) . On the other hand, the presence of dye on the right-side levels appeared to be narrower than its macroscopic distribution for both corpses. Notably, while for the corpse A, the staining involved only the adipose tissue at a distance from the vascular and nervous structures in positive samples, the same was true for the corpse B except for the T4/5 level (medial sample), for which the dye was nearby the adventitia of the vessels, even if is still far away from the intercostal nerve (Figs. 4, 5). Overall, the dye diffused ventrally to the intercostal spaces (2–3 spaces on the right and 5–6 spaces on the left) by following the blood vessels coupled to the dorsal nerve passing through the costotransverse foramen (Fig. 5).

## Discussion

The present study suggested the pattern of anterior diffusion of the local anesthetic in ESP block by gross anatomical dissection with histological examinations.

In ESP block, the mechanism of pharmacodynamics action was hypothesized to be in part due to the involvement of the dorsal rami of spinal nerves by superficial and deep spreading among muscles, and in part due to the penetration of the local anesthetic anteriorly to reach the intercostal nerves, both coherently to the distribution of cutaneous dermatomes [1, 6, 9]. Notwithstanding, even in the original description of ESP block, there was no definitive confirmation and characterization of dye spreading to involve the intercostal nerves [1], although if the paravertebral diffusion or their sporadic involvement was reported in some studies [1, 9–13], while others failed to prove it as much in humans as in pigs [5–8]. This contradictory evidence on the issue matched with the practical observation that the effectiveness of ESP block is not entirely reliable and reproducible in daily clinical practice [16].

In the present study, the number and location of the intercostal spaces showing macroscopic signs of anterior dye diffusion were approximately symmetric between both sides from T2/3–T9/10, substantially centered on the injection site according to previous data [1, 5, 7, 9–12].

Histologically, dye staining of the medial soft tissue forming the intercostal space on samples retrieved by anatomical microdissection was here demonstrated. A discrepancy was detected between the macroscopically detected coloring of the intercostal spaces, and the histological confirmation on the same sample, more evident on the right side. This finding could be explained by the possible discoloration of weakly dye-stained samples.

The overall evidence provides a further basis for the analgesic effect reported in daily clinical practice, at the same time, confirming the presence of an anatomical pathway for the anterior spread of local anesthetic [1, 17–19]. A possible pathway through the costotransverse foramen and its costotransverse ligaments was suggested [1, 9, 11, 12], as demonstrated by recent evidence [10] supporting the introduction of the costotransverse foramen block technique [20]. The costotransverse foramen (Fig. 3) is an anatomical space bordered cranially by the transverse process, caudally by the underlying rib, laterally by the medial-free edge of the superior costotransverse ligament, and medially by the lamina and facet joints [1]. The costotransverse ligament consists of both the superior costotransverse ligament and the lateral costotransverse ligament [12, 21, 22]. The medial-free edge of the superior costotransverse ligament constitutes the lateral border of the costotransverse foramen and the lateral portion of the superior costotransverse ligament joins with the internal intercostal membrane [22, 23]. The superior costotransverse ligament has anterior and posterior layers and presents gaps in its structure [21, 24, 25] (Fig. 3). At each thoracic segmental level, the dorsal ramus of the spinal nerve and the accompanying blood vessels pass through the costotransverse foramen between the anterior and posterior layers of the superior costotransverse ligament [12, 21, 23, 24] (Fig. 3). However, the hypothesis of an anterior diffusion path of the dye through the costotransverse foramen has been somehow criticized by some authors, as the complex attachment of the erector spinae muscle group to the transverse process and the costotransverse ligaments may prevent the anterior anesthetic diffusion [5, 7, 10–12].

The ventral pathway of the dye through the costotransverse foramen to reach the intercostal nerves is suggested by the dye spread in the intercostal soft tissue through the direct infiltration demonstrated at histotopographic examination. In particular, four aspects converge on this consideration. First, the macroscopic involvement of the dorsal rami of spinal nerves was detected. It is known that they pass through the costotransverse foramen. Second, the medial portion of the intercostal soft tissue is placed anteriorly to the costotransverse foramen.

Third, whenever a harvested intercostal soft tissue showed dye staining at histology, the dye was detected steadily as infiltrating its medial portion, with a variable degree of further extension of the dye laterally. Fourth, the dye was demonstrated near the adventitia of the intercostal vessels, which are initially coupled with the dorsal rami of the spinal nerves and respective blood vessels passing through the costotransverse foramen as mentioned [10, 12] (Fig. 3). As a consequence, the anterior histotopographic diffusion of dye through the erector spinae plane block resembles a paravertebral block [26, 27].

Overall, the variability of shapes and dimension of the dye spreading reported in the literature for corpses may be referred to some factors, as follows. The costotransverse ligaments showed different anatomical configuration and relationships along the vertebral column [6, 28]. Indeed, the posterior costotransverse ligament is absent/rudimentary between T1 and T6, very well developed at the T7–T10, and undetectable at the T11–T12, which could negatively influence the diffusion within the paravertebral spaces at these lower levels for dye injection at the T5 level [9, 11, 12, 21, 22]. However, other authors showed as dye injection at T10 level only shifted the area of tissue spread, without limiting it downward [7]. Also, the volume and viscosity of injected dye may play a role by generating a different pressure-driven mechanism, which differs from study to study [11]. Even the time awaited for the distribution of the injected dye before dissection may have played a role in its different distribution. In the present paper, the ESP block was performed as per daily clinical practice, for both procedures and times. In literature, the time elapsed between the injection of dye and the cadaveric dissection was widely ranging up to 2 h [5, 6, 9, 10].

There are limitations in using a cadaveric model to represent the spread of local anesthetic during real-life clinical practice. First, postmortem changes in the integrity and permeability of tissues might influence dye dispersion. Second, in the living, there may be a more extended spread of local anesthetic related to intrathoracic pressure changes during breathing, or muscle tone and muscular contraction. Third, the different placement of the patient may has a role. Fourth, the assumption that observing the dye spread one can infer the clinical effect of local anesthetic has to be demonstrated. Furthermore, in this pilot study, the procedure was performed just on two corpses and variability of observations has to be considered.

In conclusion, the present study performing an ESP block in corpses confirmed the extensive anterior spread of the dye involving the intercostal nerves, both at the macroscopic and microscopic examination. The histotopographic evidence suggests that the dye’s anterior diffusion takes place through the costotransverse foramen and seems to follow the vessels and dorsal rami of spinal nerves. The dye diffusion pattern resembles a paravertebral block, hinting at a complex action mechanism to explain the ESP block clinical effect.

Although these results do not make yet clear what the definitive answer is regarding the mechanism of ESP block, it helps to understand what its path of diffusion among the anatomical planes is and which structures interest it more anteriorly. It will be of interest to investigate the anatomo-clinical causes underlying the variable effect of the ESP block observed in the daily practice, which could be related at least in part to individual morphometric differences of the structures involved by the passage of the anesthetic.

## Supplementary Information

Below is the link to the electronic supplementary material.

Supplementary file1 (MOV 3945 KB) Real-time movie related to the ESP block procedure. A 21-Gauge, 50 mm needle was inserted with a cephalad-to-caudal direction into the posterior thoracic wall at the T5 level, to reach the respective transverse process. The visualization of a linear fluid spread that distended the fascial plane between the erector spinae muscles group and the transverse process while injecting 20 mL of diluted Black tissue marking dye confirmed the proper needle tip positioning. *T5* transverse process of the 5th thoracic vertebra, *TM*  trapezius muscle, *RMM*  rhomboid major muscle, *ESM * erector spinae muscles.
